# Challenges and Perspectives in Chemical Synthesis of Highly Hydrophobic Peptides

**DOI:** 10.3389/fbioe.2020.00162

**Published:** 2020-03-04

**Authors:** Lena K. Mueller, Andreas C. Baumruck, Hanna Zhdanova, Alesia A. Tietze

**Affiliations:** ^1^Clemens-Schöpf Institute of Organic Chemistry and Biochemistry, Darmstadt University of Technology, Darmstadt, Germany; ^2^Department of Chemistry and Molecular Biology, Wallenberg Centre for Molecular and Translational Medicine, University of Gothenburg, Gothenburg, Sweden

**Keywords:** solid phase peptide synthesis, membrane-associated proteins, native chemical ligation, conjugation, transmembrane peptide

## Abstract

Solid phase peptide synthesis (SPPS) provides the possibility to chemically synthesize peptides and proteins. Applying the method on hydrophilic structures is usually without major drawbacks but faces extreme complications when it comes to “difficult sequences.” These includes the vitally important, ubiquitously present and structurally demanding membrane proteins and their functional parts, such as ion channels, G-protein receptors, and other pore-forming structures. Standard synthetic and ligation protocols are not enough for a successful synthesis of these challenging sequences. In this review we highlight, summarize and evaluate the possibilities for synthetic production of “difficult sequences” by SPPS, native chemical ligation (NCL) and follow-up protocols.

## Introduction

The “difficult sequence” concept has been introduced in the 80’s and was given distinction by Kent and co-workers for peptides that form strong inter- or intra molecular, non-covalent interactions which form insoluble peptide aggregates. “Difficult sequences” are peptide sequences that contain high number of amino acids possessing hydrophobic side chains, so-called β-branched amino acids, including leucine, valine, phenylalanine or isoleucine. Additionally, glycine is known to induce β-sheet packing in combination with afore mentioned amino acids (Paradis-Bas et al., 2016). These sequences tend to form β-sheet or α-helical structures within the molecule and therefore they have high aggregation potential and low solubility in aqueous or organic solvents. This results in a generally difficult handling, synthesis and purification.

**FIGURE 1:**
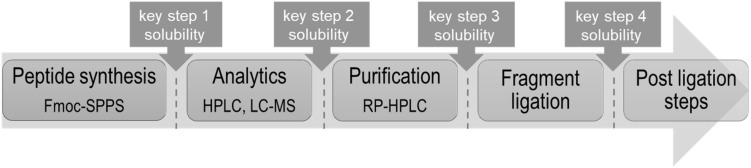
Schematic way for chemical production of highly hydrophobic peptides with highlighted key steps requiring solubility.

**FIGURE 2:**
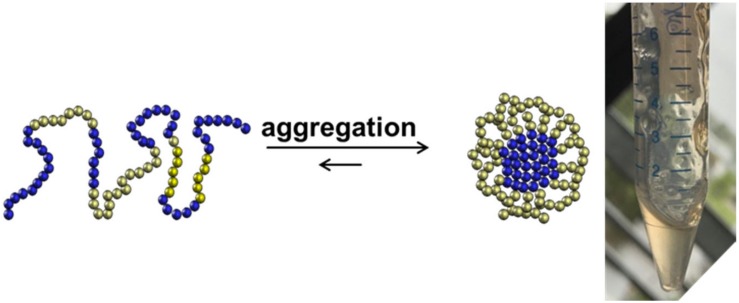
Aggregation of highly hydrophobic peptides as a challenging behavior for chemical synthesis.

One should consider that the chemical production of “difficult sequences” is composed of several key steps, which include SPPS, analytical characterization, purification, fragment ligation and if needed post ligation steps (Figure 1). Each mentioned step is challenging because of the high probability of “difficult sequences” to aggregate and precipitate in conventional solvents (Figure 2). Consequently, solubility of “difficult” peptides and proteins is needed at every key step of their production route. Last decades, researchers developed various methods to achieve this major goal. However, when screening through the literature, unfortunately no unique protocol is available for the synthesis of highly hydrophobic “difficult” peptides and proteins. Nowadays, for every new intended synthesis, special optimization of SPPS, NCL and follow-up protocols are still required.

In this review we highlight, summarize and evaluate the possibilities for synthetic production of “difficult sequences” by SPPS, native chemical ligation (NCL) and follow-up protocols.

## Success Reports in Synthesis of Transmembrane Proteins

Focusing on the class of heavy synthetically accessible proteins such as membrane proteins and considering their structural and functional divergence we will summarize in following efforts undertaken for their synthetic production. It is worth to mention that membrane proteins are encoded by 20–30% of the human genome (Wallin and von Heijne, 1998; Santoni et al., 2000; Krogh et al., 2001; Melnyk et al., 2003; Li et al., 2017). Thereupon, it is not surprising, that because of this relatively high abundance, membrane proteins are of great interest as drug targets. This can be seen in the number of available drugs that target these structures. Around 60% of all FDA-approved drugs address these structures, e.g., GPCRs or ion channels (Yildirim et al., 2007) and the interest in therapeutic applications or for the design of nanopore-based bio-inspired sensors is rising. Surprisingly, until 2003 only about 60 high-resolution structures of transmembrane proteins were available despite their great importance for the cell function and drug design (Melnyk et al., 2003). With the Nobel prize in 2017, awarded to J. Dubochet, J. Frank, and R. Henderson on their outstanding work in cryo-electron-microscopy, this situation changed rapidly (Cressey and Callaway, 2017; Allen, 2019). High profile structures, dynamics and structural studies of various membrane proteins are now available, including works on calcium-selective ion channels (Yoo et al., 2018), voltage-gated potassium channels (Shigematsu et al., 2017; Shigematsu et al., 2019) and a membrane-embedded monomeric yeast ATP synthase (Srivastava et al., 2018).

However, there is still a challenge to provide access to enough material to determine structures and functions of membrane proteins. Two major ways are possible either to chemically synthesize or recombinantly express membrane proteins.

Four major problems in expression of these structures led to the development of strategies using chemical synthesis: (1) protocols for the recombinant expression of hydrophilic structures are often not applicable to hydrophobic proteins, (2) over-expression of membrane proteins usually leads to membrane disruption and thus cell toxicity, (3) extraction and purification are not trivial, and (4) limitations in incorporation of post-translational modifications/isotopic labels (Shen et al., 2011).

Trying to overcome these problems, a bridge between biology and chemistry was required, resulting in an outstanding cooperation. A great advantage of chemical synthesis displays the possibility to custom design of the desired sequence. As soon as the successful synthetic protocol is elaborated, the integration of unnatural amino acids, mutations at arbitrary positions, post-translational modifications (PTMs) and site-specific labels for e.g., solid-state/solution NMR spectroscopy or fluorescence microscopy experiments is readily possible (Sato, 2016). Furthermore, product in the multi-milligram range can be obtained making numerous analytical experiments possible, leading to a better structural and functional understanding. The establishment of a successful chemical synthesis strategy, though, faces some challenges.

Due to the challenges in synthesis of “difficult sequences” especially membrane proteins or their functional parts, only few manuscripts report successful examples and are summarized in Table 1 identifying special features of the synthetic strategy.

**TABLE 1:** Overview of successful chemical (semi-)synthesis protocols for transmembrane proteins (extended from Shen et al., 2011).

**Name**	**Protein length**	**Protocol, SPPS**	**SPPS**	**Facilitating NCL**	**Special features**	**References**
**NS4A**, cofactor protein of serine protease from Hepatitis C virus	1–66	Fragment 1: Boc-based Fragment 2: Fmoc-based	Fragment 1: tri-lysine solubilizing tag Fragment 2: tetra-lysine solubilizing tag	β-octyl-glucoside	One of the first described synthesis routes	Bianchi et al., 1999
**BM2** proton channel, influenza A	1–97	Both fragments: Boc-based		30% TFE		Kochendoerfer et al., 1999
Potassium Channel **KcsA**	1–125	Fragment 1-73 recombinant expression Fragment 74-125: Boc-based		50% TFE, 1% SDS	Thioester Fragment 1-73, Great difficulties in solubilizing synthesized fragment, T74C mutation	Valiyaveetil et al., 2002
Mechano-selective ion channels: **Ec-MscL** and **Tb-MscL**	1–136	All fragments: Boc-based		Dodecyl-phospho-choline, DPC	Ec-MscL: Q56C and N103C mutations Tb-MscL: E102C and S52C mutations, Acm protection group	Clayton, 2005
Diacylglycerol Kinase from E. Coli, **DAGK**	1–121	Three fragments: Boc-based	Polyethylene glycol-polyamide (PPO) tag and hexa-arginine tag	DPC or OG	Several solubilizing tags were tested	Lahiri et al., 2011
Inward rectifier K+ channel protein **Kir 5.1**	64–179	Four fragments: Fmoc-based	Fragment 3: tetra-arginine tag	DPC	Hydrazides for NCL, usage of pseudoprolines	Zheng et al., 2014
Hepatitis C Virus cation-specific ion channel **p7**	1–63	Both fragments: Fmoc-based	Both fragments: tetra-arginine tag		Hydrazides for NCL, removable backbone modifications (RBM) consisting of polyargininge-tagged groups	Zheng et al., 2016
Ser64-phosphorylated **BM2** proton channel, influenza A virus	1–97	Both fragments: Fmoc-based	Solubilizing unit Arg4 (RBM)		Hydrazide mediated NCL, Arg4 tag removed at end (TFA)	Tang et al., 2017
**BM2** proton channel, influenza A virus	1–51	Both fragments: Fmoc-based	Various solubilizing tags (ADO, ADO2, ADO-Lys5)	TFE or HFIP (2:1)	Oxo-ester for NCL	Baumruck et al., 2018
Copper storage protein 1 **CSP-1**	1–122	Three fragments: Fmoc-based	Three fragments: Phacm solubilizing tag		Phacm temporary solubilizing tag, selective palladium-mediated deprotection of Thz	Jbara et al., 2018
Interferon-induced transmembrane protein 3 (**IFITM3**)	1–133	Fmoc-based	Oligo-arginine (Arg7)	95:5 NMP:H2O	KAHA ligation*	Harmand et al., 2017

*See outlook section for more information.

Evaluating the synthetic strategies, a definite shift from Boc-based synthesis to the less toxic Fmoc-based protocols from 2010 is visible which can be connected to the increasing availability of novel NCL strategies. An advancement of the hydrazide mediated NCL is noticeable with NCL yields of 39% (Zheng et al., 2016; Tang et al., 2017). However, this method is hampered by impossible incorporation of removable C-terminal solubilizing tag to the hydrazide moiety. Lately, Baumruck et al. (2018) presents an oxo-ester mediated NCL with almost quantitative NCL yields introducing an interesting prospect by attaching removable solubilizing tag to the oxo-ester. This strategy benefits from the easier handling of highly hydrophobic thioester-forming peptide due to its solubility while purification and analysis prior to NCL. Moreover, no additional steps are required to remove the solubility tag from the peptide sequence, it is automatically removed at NCL conditions. Another example is the use of removable backbone modifications which remain stable throughout NCL, facilitating the reaction, and are readily cleaved off afterward using TFA. Additionally, as seen in the table (Table 1), a preference for arginine-based solubilizing tags can be noticed. A brilliant example of using an arginine tag [phenylacetamidomethyl (Phacm) attached to cysteine] and selective orthogonal removal of the Cys masking group 1,3-thiazolidine-4-carboxylic acid (Thz) without affecting this tag was demonstrated by Jbara et al. (2018). Moreover, most of the presented synthetic strategies include addition of organic solvents such as TFE (Kochendoerfer et al., 1999; Valiyaveetil et al., 2002; Baumruck et al., 2018) or surfactants like OG and DPC (Bianchi et al., 1999; Clayton, 2005; Lahiri et al., 2011; Zheng et al., 2014) to the ligation solution. Finally, Tang et al. presented a synthesis and ligation route toward BM2 that was published as a protocol, establishing a basis for generation of general approach.

**FIGURE 3:**
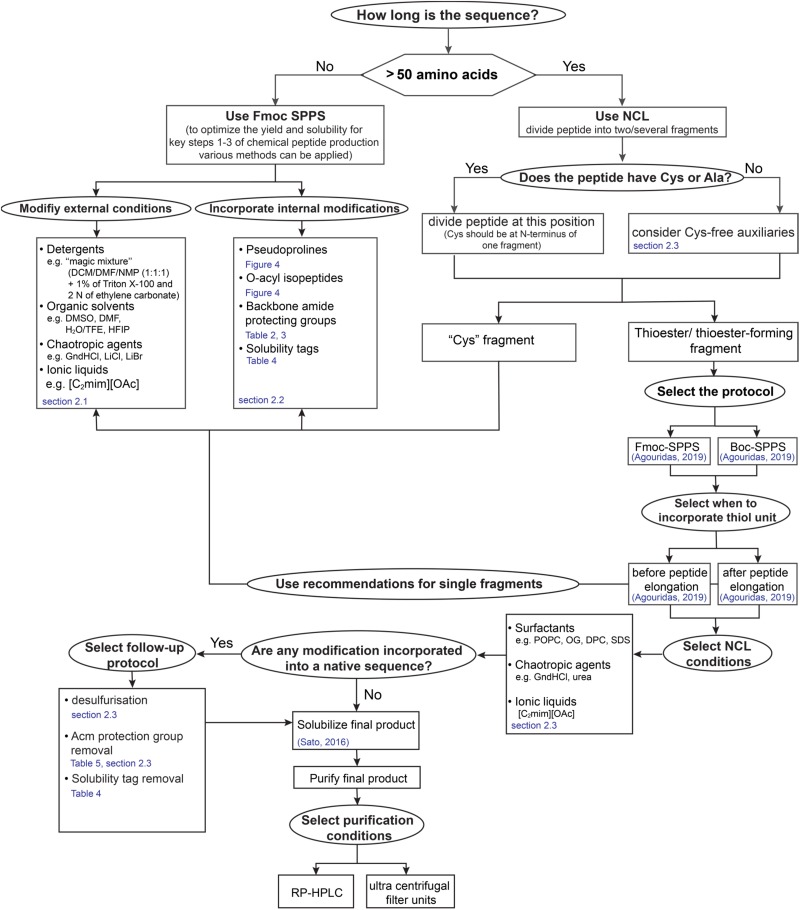
“Yes-no” diagram for making decisions in the process of “difficult sequences” chemical production.

The successful synthesis of highly hydrophobic sequences represents the feasibility of the strategies developed during last decades. However, divergence of the methods does not give a standard recipe for scientists on which method is the most applicable for the chemical production of novel sequences. Therefore, we designed a “yes-no” diagram which is intended to facilitate decisions at each production step and in following chapters we highlight mentioned methods in more detail (Figure 3).

## Solid Phase Peptide Synthesis and Native Chemical Ligation of “Difficult Sequences”

The introduction of solid phase peptide synthesis (SPPS) in 1963 by Merrifield revolutionized the former state-of-the-art liquid phase strategies and made synthesis of peptides and proteins easier, more efficient and accessible (Merrifield, 1963, 1969). Within the next years, this strategy was continuously improved but it was only in 1970 that Carpino and Han transformed the working scheme by introduction of Fmoc-based strategy (Carpino and Han, 1970) making this the more advantageous and less toxic version of SPPS with respect to usage of hazardous hydrofluoric acid (HF) (Palomo, 2014; Jaradat, 2018). This improvement in reaction conditions led to a rise in prosperous synthesis of peptides following the Fmoc-protocol, making automated synthesis applicable. However, peptide chains of “difficult sequences” exhibiting over 50–60 amino acids remain a challenge even when applying automated peptide synthesis protocols (Kochendoerfer and Kent, 1999; Sheppard, 2003; Palomo, 2014; Behrendt et al., 2016). Microwave assistance is usually advantageous to improve the yield of the final peptide, which however reaches its limits while synthesizing “difficult sequences” (Erdelyi and Gogoll, 2002; Paradis-Bas et al., 2016).

The last decades have witnessed an unprecedented progress in chemical peptide synthesis, however there were still sequences which were difficult or even impossible to synthesize by standard SPPS belonging to class of “difficult sequences,” i.e., amylin (Cooper et al., 1987; Harris et al., 2013), Aβ(1-42) (Bacsa et al., 2010; Kasim et al., 2019), and BM2(1–51) (Baumruck et al., 2018). With respect to SPPS “difficult sequences” are defined as peptides that are poorly solvated while attached to the solid support thus preventing complete deprotection and coupling steps (Tickler and Wade, 2007). This “on resin aggregation” is often associated with sequences that contain β-sheets or α-helices which are mostly represented in membrane-associated peptides/proteins. The type and loading capacity of the resin support has a tremendous influence on the quality of the crude peptide. For hydrophobic peptides non-polar resins such as polystyrene proved to result in peptide products with high quality, yield and purity compared to peptides synthesized on polar resin such as polydimethylacrylamide (Tickler and Wade, 2007). This finding can be explained by the minor aggregation potential of hydrophobic amino acids sequences on polar resins. For the Boc-based SPPS protocol one of the first strategies which is used to optimize involves the acetylation of the imino-group of histidine and was introduced in 1966 by Weygand, subsequently being used by others (Weygand et al., 1966b). Oliveira et al. (1997) investigated the impact of the solvent during coupling. Using DMF, they received a yield of 4% when synthesizing a hydrophobic transmembrane 34-residue peptide fragment of the rat bradykinin receptor. By changing the solvent to 80% NMP/DMSO increased coupling yields (12%) were observed due to improved swelling of the benzhydrylamine (BHAR) resin (loading: 0.34 mmol/g), while testing the Fmoc-based SPPS, no product was obtained.

In general, Boc-based SPPS is known to show better results for the synthesis of “difficult sequences” compared to the Fmoc-based strategy (Schnolzer et al., 2007; Dittmann and Martin, 2017). This is, on one hand, ascribable to TFA, which selectively dissolves the protected peptide chain during SPPS and disrupts formations of secondary structures (Schnolzer et al., 2007). On the other hand, optimizations of Boc-based SPPS protocols using *in situ* neutralization protocols, favors synthesis of difficult sequences as well. Treatment with TFA leads to the formation of α-ammonium species that needs to be neutralized prior to coupling, but when neutralized, leads to aggregation. Using an *in situ* protocol, a high concentration of activated amino acid in a polar solvent containing DIEA is added directly, thus minimizing aggregation (Alewood et al., 1997; Schnolzer et al., 2007). One of the limitations using Boc-based SPPS is caused by the continuous use of strong acid during and cleavage from the resin with HF though. Therefore, Boc-based SPPS is not suitable for backbone modifications designed for Fmoc-based SPPS. Nevertheless, Johnson and Kent introduced a photolytically cleavable 4-methoxy-2-nitrobenzyl (2-Nb) and 4-methoxy-2-nitrobenzyl (4-OMe-2-Nb) backbone amide protection groups, illustrated on a model peptide MG(X)GFL (X = 2-Nb or 4-OMe-2-Nb) that can be introduced for the synthesis of “difficult sequences” using the Boc-based protocol (Johnson and Kent, 2006).

With the rising interest, especially regarding therapeutic and pharmaceutical research, a way to chemically synthesize longer peptides was needed since proteins feature 250 amino acids at an average (Kochendoerfer and Kent, 1999; Kimmerlin and Seebach, 2005). To generate an amide bond in solution one must go back to 1953, when Wieland et al. (1953) made use on an intramolecular acyl shift for peptide bond formation. This method was adapted and intensively studied by Kemp and co-workers, laying the foundation of todays’ ligation strategies to fuse two or more peptide fragment (Kemp and Kerkman, 1981; Kemp et al., 1981). NCL is the method of choice for the generation of longer sequences (>50 amino acids) out of two or more fragments and was influenced by the work of Dawson et al. (1994) and Agouridas et al. (2019). At the same time, it decreases limitations of SPPS due to synthesizing shorter peptide fragments and fusing them to yield the native peptide sequence after purification and characterization of each fragment. The basic principle behind the NCL is the reaction of a N-terminal cysteine of one peptide fragment with a C-terminal thioester of another peptide fragment in aqueous phosphate buffers, containing 6 M guanidinium HCl or 8 M urea together with a reducing agent like TCEP or DTT (Dawson and Kent, 2000). However, the greatest obstacle for the NCL of lipophilic peptides is their insolubility in conventional ligation buffers. Last decades, researchers tried also to bypass the Boc-based SPPS protocol that had to be used for the synthesis of the thioester fragment.

Various strategies have been developed to improve SPPS/NCL protocols and to overcome aggregation and limitations of these methods for “difficult sequences” (Paradis-Bas et al., 2016). These methods can be divided into two main groups: (1) modifications of external conditions and (2) internal modifications of the peptide side chain or backbone (Figure 3). In following detailed strategies for optimization of SPPS and NCL for “difficult sequences” will be discussed.

### External Conditions

The addition of *organic solvents* is one of the earliest strategies to dissolve hydrophobic peptides. Polar organic solvents like DMF, DMSO, and NMP are known for their increased solvation potential to inhibit peptide aggregation on the resin. A so-called “magic mixture,” which is composed of DCM, DMF and NMP (1:1:1) has become famous for the synthesis of hydrophobic peptides and was successfully applied for the synthesis of various “difficult sequences” (Tickler and Wade, 2007). Similarly, for the NCL these solvents also found their application as additives to conventional ligation buffers. For example, the NCL of transmembrane peptides such as the rhodopsin II/transducer complex was performed in the presence of DMSO or DMF resulting in 65% yield (Dittmann et al., 2010, 2014). This strategy was also successfully applied for the ligation of various other hydrophobic proteins, such as small hemithioindigo (HTI)-based chromopeptide (Kitzig and Ruck-Braun, 2017), and O-acyl isopeptides (Sohma et al., 2011).

Another promising approach is the addition of 2,2,2-trifluoroethanol (TFE) or 1,1,1,3,3,3-hexafluoro-2-propanol (HFIP) to N,N-dimethylformamide (DMF) during the coupling steps in order to increase the polarity and solvation properties of the solvent. Examples for this strategy represent the synthesis of d-Ala^17^-*phGnRH*(14–36) (Milton and Milton, 1990) or model “difficult sequences” (Yamashiro et al., 1976). Fluorinated alcohols are also known as effective solvents for hydrophobic peptides, which can be used during analytics and purification, namely key steps two and three, of the chemical production (Figure 1; Tiburu et al., 2009; Bondarenko et al., 2010; Zhang et al., 2018). The reason for that is that TFE or HFIP mimic the natural environment of the cell membrane thus mediate the dissolution of membrane-associated peptides. Intra-molecular H-bonds can be stabilized through fluorinated alcohols, preserving especially the α-helical structure of the transmembrane region (Hirota et al., 1996). Fluorinated alcohols such as TFE, HFIP or 1-phenyl-2,2,2-trifluoroethanol (PhTFE) also found their application in NCL as co-solvent. Impressing examples of this approach were published by M. Hong et al. and W. DeGrado et al. who used TFE and HFIP as co-solvents for the NCL during the synthesis of influenza virus A proton channel AM2 (Kochendoerfer et al., 1999; Kwon et al., 2015) and in our studies for the synthesis of influenza virus B proton channel BM2(1–51) fragment (Baumruck et al., 2018).

*Chaotropic agents* belong to a group of water-soluble ingredients which can disturb hydrogen bonds between water molecules and proteins. Examples for chaotropic agents include various salts, detergents, polar organic solvents or urea and thiourea. Additionally, chaotropic ions such as Li^+^, Mg^2+^, SCN,^–^ and ClO_4_^–^ can be added to reduce non-covalent hydrophobic interactions and consequently prevent precipitation of poor-soluble peptides (Dawson et al., 1994; Zhang and Cremer, 2006).

The effect of ions on the stability of secondary and tertiary structures and on the solubility of peptides were intensively studied by multiple groups (Dawson et al., 1994) and summarized in the Hofmeister series (Cacace et al., 1997). The use of chaotropic agents is of great importance for NCL reaction with the first reported NCL studies by Kent and co-workers already performed in guanidinium hydrochloride-based buffers (Dawson et al., 1994). Nowadays, the majority of ligations are executed in aqueous buffers containing high concentrations of chaotropic agents like urea or guanidinium hydrochloride (Paradis-Bas et al., 2016). These two ingredients prevent the formation of unfavorable secondary structures thus enhancing the solubility of peptides. However, ligation of hydrophobic peptides is strongly limited in these standard ligation buffers due to the poor solubility of the peptide/protein fragments.

In contrast to chaotropic agents, *surfactants* are applied to mimic the natural environment of cell membranes. In aqueous solutions surfactants form micelles or lipid liposomes in which hydrophobic transmembrane peptides can easily be incorporated. Several studies demonstrated that transmembrane proteins spontaneously fold while incorporated in an artificial lipid membrane. This behavior is utilized in analytical applications such as circular dichroism (CD) spectroscopy and solution nuclear magnetic resonance (NMR) studies (Engelman and Steitz, 1981; Ladokhin et al., 2010; Mandala et al., 2019). In order to aid NCL multiple surfactants were investigated to increase the solubility of transmembrane peptides in guanidinium chloride or urea as additives to ligation buffer. Main advantage accompanied using surfactants is their commercial accessibility and fast experimental screening. Examples for frequently used surfactants are 1-palmitoyl-2-oleoylphosphatidylcholine (POPC) (Otaka et al., 2004), n-octyl glycoside (OG) and dodecylphosphocholine (DPC) (Lahiri et al., 2011). An impressive example for the value of this strategy was made by Becker and co-workers who used n-octyl glycoside (OG) as an additive for the NCL to synthesize transmembrane peptide diacylglycerol kinase (DAGK) comprising 121 amino acids (Lahiri et al., 2011). Other successful examples were reported using SDS for the NCL of the potassium channel KcsA comprising 125 amino acids (Valiyaveetil et al., 2002). Although surfactants are applied as additives for protein and peptide solubilization for many years, several publications indicate that they could interfere with RP-HPLC columns and reduce the signal intensity in mass spectrometry (Loo et al., 1994; Paradis-Bas et al., 2016).

During the last decade *ionic liquids* (ILs) gathered much attention as potent reaction and solubilizing media for biomaterials, including hydrophobic peptides and cellulose (Swatloski et al., 2002; Miloslavina et al., 2009). Several promising examples were published where ILs, in particular 1-ethyl-3-methylimidazolium acetate ([C_2_mim][OAc]) was described as advantageous solvent for oxidative folding of highly hydrophobic cysteine-rich peptides (e.g., conotoxins) or NCL (Miloslavina et al., 2009; Tietze et al., 2012; Böhm et al., 2014). The [C_2_mim][OAc] was used as media for the NCL of the 66-meric tridegin, which is known as an inhibitor of human blood coagulation factor XIIIa (Böhm et al., 2012). However, N-heterocyclic carbenes (NHC) which are present in the neat [C_2_mim][OAc], generated by subtraction of C2 proton by relatively basic acetate anion, initiated formation of a high percentage of by-products and therefore prevented the breakthrough of this method. In 2017, we identified those NHC-induced side-products and studied conditions where the formation of those side-products can be entirely suppressed, therefore enabling using [C_2_mim][OAc] as a solvent during NCL (Baumruck et al., 2017). Additionally, Wehofsky et al. (2008) reported protease-catalyzed ligation in the presence of 60% of 3-methylimidazolium dimethylphosphate in buffer solution with high turnover rates.

### Internal Modifications

*Temporary internal (structural) modifications* of the peptide sequence have revolutionized the use of the Fmoc-based SPPS protocol on “difficult sequences.” Today several different backbone modifications exist mainly focused to prevent side-reaction such as diketopiperazine or aspartimide formation (Paradis-Bas et al., 2016). Many of them offer also the advantage to reduce peptide aggregation on solid support thus facilitate the accessibility of the N-terminus during SPPS (Tickler and Wade, 2007). Depending on their structure, most backbone modifications can be classified in ortho-hydroxybenzyl groups [Hmb (Hyde et al., 1994), Hmsb (Huang and Liu, 2015), Hbz (Johnson and Quibell, 1994), Hnb (Meutermans et al., 1999)] ortho-mercaptobenzyl groups [Dmmb (Kawakami et al., 2001), Tmb (Offer et al., 2002)] and methoxybenzyl groups [Dmb (Zahariev et al., 2006), Mmsb (Thennarasu and Liu, 2010)]. Additionally, several other protection groups were developed which cannot be assigned to one of the aforementioned backbone modifications such as EDOTn (Isidro-Llobet et al., 2008), Dcpm (Carpino et al., 2009), Etom (Fernandez-Llamazares et al., 2014), 2-furfury and 2-thlenylmethyl (Johnson et al., 1995) groups. In the 1990ies, one of the first backbone amide protection groups, which remarkably improved handling of aggregation-prone peptides, was developed, N,O-bis(Fmoc) derivates of Nα-2-hydroxy-4-methoxybenzylamino acids (Hmb) (Zeng et al., 1997). This group demonstrated the ability to facilitate synthesis of an acyl carrier protein, a 65–74 decapeptide that showed strong inter-chain association (Weygand et al., 1966b; Quibell, 1999). Thus, the Hmb moiety and its derivates were developed, which were frequently used in Fmoc-based SPPS (Simmonds, 1996; Table 2).

**TABLE 2:** Backbone amide protecting groups that can be removed during cleavage.

**Structure**	**Advantages**	**Limitations**
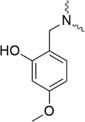 Hmb (Johnson et al., 1993, 1995) (Fmoc SPPS)	• Inhibition of aspartimide formation • Commercial availability of dipeptides and amino acids containing Hmb group	• Poor *O-N*-acyl transfer
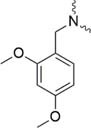 Dmb (Weygand et al., 1966a; Blaakmeer et al., 1991) (Fmoc/Boc SPPS)	• Suppression of aspartimide formation • Rapid removal at high concentrations of TFA • Commercial availability of dipeptides and amino acids containing Dmb group	• Bulkiness • Dipeptides are restricted to Substitution sites containing Ser, Thr and Gly (and in some cases Cys)
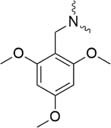 Tmob (Johnson et al., 1995) (Fmoc SPPS)	• Commercial availability of Tmob-protected amino acids • Faster acylation compared to Dmb. • High acid lability, can be removed with 5% TFA in DCM	• Bulkiness
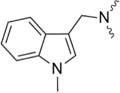 MIM (Isidro-Llobet et al., 2008) (Fmoc SPPS)	• Lower steric hindrance and faster acylation compared to Dmb	• Bulkiness
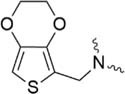 EDOTn (Isidro-Llobet et al., 2008) (Fmoc SPPS)		
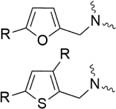 2-furfury/2-thienylmethyl (Johnson et al., 1995) (Fmoc SPPS)	• Higher acid lability compared to Hmb	• Inefficiency of the coupling of the incoming amino acid onto the N-(furfury/thienyl)peptidyl-resin

**FIGURE 4:**
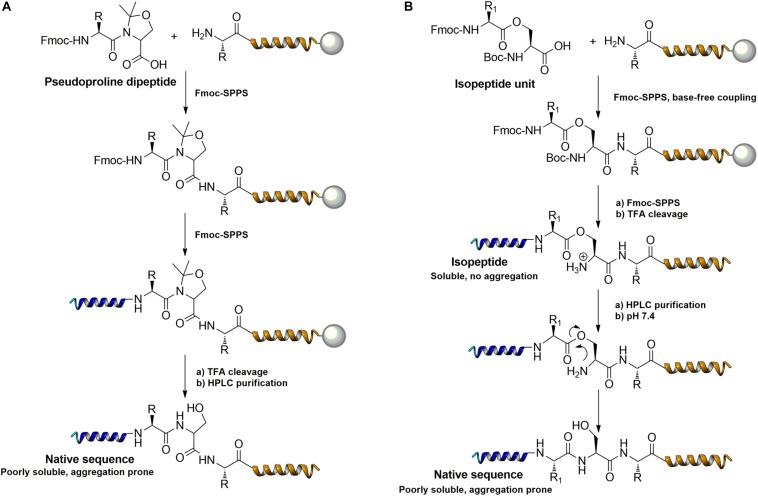
Example of pseudoproline **(A)** and isopeptide **(B)** strategies as temporary structural modifications.

Further strategies aiming to decrease on-resin aggregation during SPPS include the use of pseudoprolines (Mutter et al., 1995; Wohr and Mutter, 1995) or O-acyl isopeptides (Horikawa et al., 1998) (depsipeptides) (Figure 4). Mutter’s lab introduced pseudoprolines incorporating them into a sequence with numerous building blocks being commercially available (Mutter et al., 1995; Wohr and Mutter, 1995). The cyclic oxazolidine (Ser, Thr) thiazolidine (Cys) ring system shows structural similarities with Pro, resulting in a “kink” conformation within the growing peptide chain preventing aggregation, self-association and a β-sheet formation (Wohr et al., 1996). An alternative strategy to interrupt unfavorable secondary structures is the synthesis with O-acyl isopeptides which were developed by Sohma et al. (2011). This strategy relies on the introduction of oxo-esters over Ser or Thr residues within the primary sequence. The strategy was successfully applied for SPPS of several lipophilic peptides (Kawashima et al., 2014).

Apart from their structure, *backbone modifications* can be classified into two categories: (1) modifications, removable during cleavage from the resin (Table 2) and (2) modifications, removable after cleavage from the resin (Table 3).

**TABLE 3:** Backbone amide protecting groups that remain on the sequence after cleavage from solid support and require special conditions to be detached.

**Structure**	**Advantages**	**Limitations**
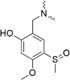 Hmsb (Howe et al., 2000) (Fmoc SPPS)	• “Safety-catch” protecting group	• Bulkiness
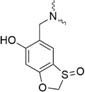 1,3-Benzoxathiole-3-oxide derivative (Offer, 1997) (Fmoc/Boc SPPS)	• Efficient acylation, suppression of epimerization • “Safety-catch” protecting group	• Bulkiness
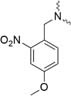 4-Methoxy-2-Nb (Johnson and Kent, 2006) (Fmoc/Boc SPPS)	• “Safety-catch” protecting group	• Slightly slower coupling of incoming amino acids onto the 4-methoxy-2-Nb-peptidyl-resin compared to Hmb
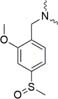 Mmsb (Paradis-Bas et al., 2014) (Fmoc SPPS)	• Resistance under acidic conditions and acidic lability after the reduction of sulfoxide • Prevention of aspartimide and diketopiperazine formation • Facilitation of cyclization	• Bulkiness

The first group of modifications only facilitates the SPPS by preventing on-resin aggregation of “difficult sequences” (Table 2). Although, the second group, includes modifications which are stable during final cleavage from solid support and additionally improves the handling and purification of poor-soluble peptides after cleavage from the solid support (Table 3).

Lately, the use of solubilizing units/tails/tags gained great attention. These units are usually made of multiple hydrophilic amino acids that are attached to the hydrophobic sequence and are apparently designed toward facilitating purification *via* HPLC, peptide condensation or NCL. Especially the use of polyarginine and polylysine units is widely represented in literature (Zheng et al., 2014). *Solubilizing tags* can be attached to the N-terminus, C-terminus or side chain of amino acids in a sequence. *N-terminal tags*, are barely represented in literature and mostly used to increase the solubility of hydrophobic peptides for purification. Examples for N-terminal tags were reported for different model peptides (Englebretsen and Robillard, 1999; Tsuda et al., 2018b) and for the insulin A chain (Disotuar et al., 2019). *C-terminal tags*, belong to the most applied strategies to increase the solubility of poorly soluble peptides. In Englebretsen and Alewood (1996) were the first who tested a C-terminal poly-glycine-arginine tail to increase the solubility of the peptide CP10 (42–55). All these first approaches were mainly used to aid the purification of hydrophobic peptides *via* HPLC. Further examples of similar strategies were published for insulin glargine (Hossain et al., 2009), NY-ESO-1 (Harris and Brimble, 2010) or Q11 (Paradis-Bas et al., 2015). Especially useful is the C-terminal tag strategy in combination with MPA-thioester peptides (3-mercaptopropionic acid, MPA). Thereby, the solubilizing tag can be directly attached at the MPA moiety which is removed during NCL. Successful examples for this method were reported for the ligation of HIV 1 protease (Johnson et al., 2007), ORL1 (288–370) (Sato et al., 2005), DEN2C (21–100) (Zhan et al., 2013), and many more (Sohma et al., 2008; Yang et al., 2013). However, major disadvantage of MPA-thioester peptides is their compatibility with Boc-based SPPS. A useful alterative to MPA-thioester peptides are Dbz and MeDbz groups which are compatible with Fmoc-based SPPS. This novel approach was used for the ligation of SUMO-2 and ubiquitin (1–93) (Bondalapati et al., 2017). In 2018, we introduced another Fmoc-based SPPS compatible C-terminal tag (polylysine, miniPEG) strategy using thioester-forming oxo-esters by introduction of 2-hydroxy-3-mercaptopropionic acid (Hmp) group for the ligation of the proton channel M2 of influenza virus B resulting in almost quantitative ligation yield (Baumruck et al., 2018).

Beneath C-terminal solubilizing tags the second most applied method is the attachment of *side-chain or backbone tags* to hydrophobic peptides. *Side-chain tags*, can be introduced during or after sequence elongation and after the fragment is prepared. A recent publication by Tsuda et al. (2019b) described a method using trityl-Lys (Trt-K) and trityl-Arg (Trt-R) solubilizing tags that can be inserted directly to the unprotected peptide and thus serves as an aid during NCL. They were able to successfully synthesize Cp149-NH_2_, a Hepatitis B capsid protein out of three fragments. A further recently designed method was presented by Brailsford et al. (2018) who introduced a novel arginine-modified acetamidomethyl tag (Acm^R^). Making use of the orthogonal Cys protection group Acm, which remains bound during final cleavage and removed in an additional step using mild reducing conditions. Here, the acetyl group is functionalized by introduction of polyarginine residues. The successful synthesis of a β-subunit of the human thyroid-stimulating hormone highlights the applicability of this method. A further way of utilizing a side chain solubilizing unit coupled to a cysteine moiety was recently presented by the Brick group. They introduced a phenylacetamidomethyl tag (Phacm) containing three arginine units and efficiently synthesized the copper storage protein CSP-1 (Jbara et al., 2018). Examples of solubilizing tags and even combination of both strategies – removable backbone modifications and solubilizing tags – are given in the Table 4 (reviewed in detail by Zhao et al., 2019).

**TABLE 4:** Examples of combinations of removable backbone modifications and solubilizing tags.

**Structure**	**Features**
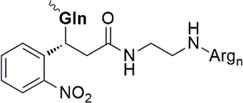 Nitrobenzene linkage solubilizing tag (Huang et al., 2013)	• Attached to Gln • Synthesis of the tag: three steps, 51% yield • Introduced during SPPS • Selective complete removal (365 nm UV) • Cannot be used for synthesis of the esterified proteins
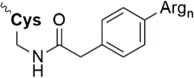 Phacm linkage solubilizing tag (Maity et al., 2016a)	• Attached to Cys • Synthesis of the tag: four steps, 35% yield • Introduced during SPPS • Selective complete removal (MgCl_2_/PdCl_2_, 6 M Gdn HCl)
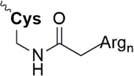 Acm linkage solubilizing tag (Brailsford et al., 2018)	• Attached to Cys • Synthesis of the tag: two steps, 28% yield • Introduced during SPPS; • Deprotection under the typical conditions of Acm removal; no additional steps needed
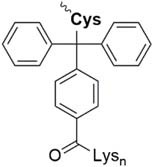 Trt-K/R_n_ solubilizing tag (Tsuda et al., 2018a)	• Attached to Cys • Synthesis of the tag: 56% yield • Must be introduced after the cleavage of the fragment from the resin • Low removal efficiency (TFA/TIS, 41%)
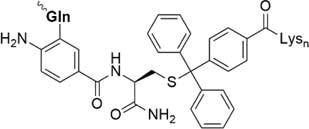 Dbz-Trt-K_n_ solubilizing tag (Tsuda et al., 2019a)	• Attached to Asp, Glu, Asn, Gln • Synthesis of the tag: four steps, 78% yield • Must be attached after the fragment is prepared • Removal: NaNO_2_, then hydrolysis or ammonolysis
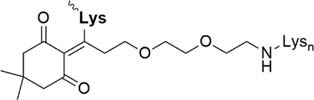 Ddae linkage solubilizing tag “helping hand” (Jacobsen et al., 2016)	• Attached to Lys • Synthesis of the tag: one step, 67% yield • Introduced during SPPS • Can be directly removed after NCL or desulfurization (1 M hydrazine)
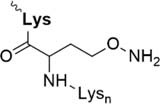 Can linkage solubilizing tag (Tsuda et al., 2018b)	• Attached to Lys • Synthesis of the tag: five steps, 21% yield • Introduced during SPPS • Hydrazide method of peptide thioesters preparation cannot be applied if Can-tag is used since the Can residue may react with NaNO_2_ • Desulfurization of Can-containing peptide is challenging • Removal 1 M NH_4_OAc (pH 4.5)
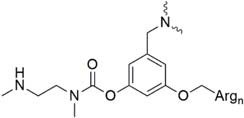 Backbone Hmb linkage solubilizing tag (Zheng et al., 2014)	• Attached to backbone of the different amino acids • Synthesis of the tag: one step, 76% yield • Despite of bulkiness, efficient incorporation into the various amino acid sites (Phe-Ile, Ile-Leu, Cys-Ile, Lys-Leu) • Inefficient incorporation into the high steric hindered sites (Val-Ile) • Prevents aggregation of the peptide • removal: TFA
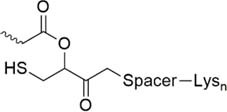 Hmp ADO, Hmp ADO_2_ or Hmp ADO-Lys_5_ (Baumruck et al., 2018)	• Attached to C-terminus • Synthesis of Hmp group: two steps, 67% yield • Convenient attachment during SPPS • Hmp is unstable in piperidine 2-methyl-piperidine must be used instead • The tag is removed during NCL
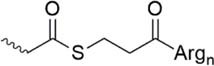 Thioester linkage solubilizing tag (Sato et al., 2005)	• Attached to C-terminus • No need to prepare a linkage in advance • Facile introduction during SPPS • Removal of the tag *in situ* during NCL • Suitable for Boc-based SPPS only • Cannot be applied for the synthesis of acid-sensitive post-translational modifications • The tag is automatically removed during NCL
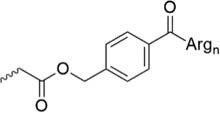 HMBA linkage solubilizing tag (Harris and Brimble, 2009)	• Attached to C-terminus • Suitable for Boc- and Fmoc-based SPPS • Easy efficient synthesis of the linker • The tag is removed during NCL

### Follow-Up Protocols

Synthesis and NCL of highly hydrophobic peptides and proteins is usually not straight-forward and requires several tools. The earlier presented solubilizing units are only one factor of several to facilitate the complete synthesis. In the following, methods will be presented that provide either the appropriate ligation side or to mask native cysteines within the sequence that are not used as ligation sites. Cysteines are not highly abundant within sequences of highly hydrophobic peptides/proteins, such as membrane proteins, necessitating incorporation of a reversable mutation within the peptide sequence. Furthermore, sequences of > 50 amino acids often need to be divided into two or more fragments. This approach though, requires masked ligation sites to enable the second or third ligation.

The classical NCL by Kent and colleges can only be performed if Cys-residue is located at the ligation site. Unfortunately, the Cys is underrepresented in most native peptide sequences with an occurrence of only 2.26% in mammals (Miseta and Csutora, 2000). To circumvent this issue of not having a Cys-residue within the sequence or at the desired ligation site, other amino acids must be found that can replace a cysteine residue and readily converted in the natural occurring moiety. The mild desulfurization of Cys to Ala residues, which are much more frequently represented in native sequences, expanded the limitations of the NCL (Pentelute and Kent, 2007). Further, the Payne lab and other groups introduced new proteinogenic amino acids aside from Ala, utilizing Asn (Sayers et al., 2015), Asp (Thompson et al., 2013), and Glu (Cergol et al., 2014) at a ligation site. Canne et al. (1996) introduced another way of circumventing the cysteine moiety by auxiliarated NCL introduced with the N^α^ -(ethanethiol) and N^α^ -(oxyethanethiol)-peptide. In Loibl et al. (2018) applied this protocol for cleavage of 2-mercaptoethyl auxiliary group. A recently introduced NCL approach, the diselenide-selenoester ligation (DSL) opens up possibilities for using further amino acids and will be elucidated in the outlook.

The earliest reports about peptide desulfurization include metal-related catalysts such as Raney nickel or palladium/aluminum oxide (Yan and Dawson, 2001). However, the usage of these metals have had some drawbacks regarding the yield, epimerization and the incompatibly with the cysteine masking group L-thiazolidine-4-carboxylic acid (Thz). That is why this method was developed further by Wan and Danishefsky, who introduced the metal-free desulfurization using TCEP and VA-044 (2,2′-azobis(2-(2-imidazoline-2-yl)propane)dihydrochloride) (Wan and Danishefsky, 2007). This widely applied method is usually performed in aqueous 6 M guanidinium hydrochloride buffer which can also be used for NCL. Nowadays, this metal-free approach is the most widely used method for NCL-desulfurization protocol and was successfully applied for multiple peptides (Jin and Li, 2018). However, despite all advantages, poor soluble peptides remain an issue due to their insolubility in conventional buffer systems. For a complete desulfurization, hydrophobic peptides need to be entirely dissolved in the buffer solution. Comparable to NCL, internal and external conditions exist to circumvent solubility problems during desulfurization. Examples for internal strategies were published by Tsuda et al. (2018a) using a side-chain polylysine tag to increase the solubility during desulfurization. Other examples include the addition of a polyarginine tag at the side chain of amyloid-beta (Aβ) peptides (Zuo et al., 2016) or polylysine tags over a Ddae-linker for the synthesis of the 97-residue co-chaperonin GroES (Fulcher et al., 2019). External conditions are usually based on the addition of co-solvents such as fluorinated alcohols to the desulfurization buffer. In 2018 we reported the use of HFIP as ideal co-solvent for desulfurization of [Cys^22^]BM2(1–51) with a yield of 99% (Baumruck et al., 2018). A further promising approach offers ionic liquids as possible media for peptide desulfurization. Studies performed in [C_2_mim][OAc] gave first evidence that Cys can be desulfurized to Ala within the sequence of unprotected peptides (Baumruck et al., 2017).

When applying desulfurization conditions, all other present cysteines need to be orthogonally protected. For that, the most common protection group is the acetamidomethyl (Acm) group proposed in 1971 and is applicable to both Boc- and Fmoc-based SPPS (Veber et al., 1968; Veber et al., 1972). To retain the native sequence, cleavage of the Acm-group needs to be performed. Table 5 gives an overview of the various, diversified methods to remove the Acm-group, with many methods also aiming for the generation of distinct disulfide-bridges within peptide sequences. Therefore, the mentioned in Table 5 methods can be applied for “difficult sequences.”

**TABLE 5:** Various protocols for the removal of the Acm protection group.

**No.**	**Acm-group removal conditions**	**Peptide synthesized**	**Product**	**References**
1	Silver trifluoromethansulfonate (AgOTf) in presence of anisole/precipitated in ether and treated with DTT	Oxytocin, chicken calcitonin	Free thiol	Fujii et al., 1989
	AgOTf followed by DMSO/aqueous HCl treatment	Tachyplesin I, endothelin I		Tamamura et al., 1995
	(AgOTf)/HCl DTT	Gstl protein		Saporito et al., 2006
2	DMSO-TFA oxidation in presence of free sulfhydryl groups	Oxytocin, human calcitonin gene-related peptide	Disulfide	Koide et al., 1993
3	Thallium trifluoroacetate, (CF3COO)3Tl in TFA	Oxytocin, human calcitonin gene-related peptide, urothensin II	Disulfide	Fujii et al., 1987
4	Iodine/acetic acid in HOAc, HCl	Human insulin-like peptide 3 analogous	Disulfide	Zhang et al., 2008
5	Triisopropylsilane in TFA (2/98)	Model peptides	Disulfide/free thiol	Ste Marie and Hondal, 2018
6	Palladium, Pd(II) complex	Ubiquitin-like protein, UBL-5	Free thiol	Maity et al., 2016b

Another, so-called “masking” group for cysteine is Thz-group, mentioned earlier in this section. When NCL must be performed in multiple steps, the Thz group helps to reversibly protect N-terminal cysteine. This group can be cleaved afterward using methoxyamine⋅HCl at pH 4 (Monbaliu and Katritzky, 2012). An additional benefit of the Thz conversion is the suitability to perform the transformation within the ligation buffer following a one-pot approach (Bang and Kent, 2004; Fauvet et al., 2013). Therefore, to use Thz in the chemical synthesis strategy followed by NCL ligation conditions must be optimized and described above. Targeting a faster unmasking reaction (usually 8 h with methoxyamine), the Brik lab introduced an extremely fast (15 min) palladium-based Thz conversion presenting efficient synthesis of Lys34-ubiquitinated histone protein, H2B (Jbara et al., 2016).

Additionally, another interesting approach was currently employed to synthesize the H2B protein (Jbara et al., 2014). The solid phase chemical ligation (SPCL) makes on-resin NCL possible and, with respect to desulfurization, is advantageous because reactants that would interfere and decrease the efficiency are washed off, e.g., MPAA.

## Hot Topics and Outlook

The great potential and importance of membrane proteins in elucidation of their structures and function as well as development of novel drug leads targeting membrane proteins causes research in this area to flourish. Moreover, need in being able to incorporate post-translational modifications, isotopic labels or peptide-mimetics rely on robust approaches of chemical synthesis (Zheng et al., 2016).

Schmidt et al. (2017) recently summarized the possibilities of ligases to perform amide bond formation during ligation reactions in a review opening a platform for biological engineering. In a very recent review, Nuijens et al. (2019) discuss advantages and disadvantages of enzymes used for ligation and cyclization suggesting a sortase-mediated ligation strategy to be straightforward. They also elucidate the possibility to use enzymes for cyclization and labeling, showing the versatile applications of engineered and naturally occurring enzymes.

With great interest to solve solubility problems during NCL, but also handling of peptides, ionic liquids seem to have enormous potential (Tietze et al., 2012; Baumruck et al., 2017). Besides SPPS and NCL, purification of these “difficult sequences” is not trivial. Apart from the usage of the already mentioned alternative organic solvents application at RP-HPLC to purify fragments and products, just recently an alternative approach has been introduced by using catch-and-release purification method which is based on base-labile cleavable linkers using oxime-based and hydrazine-based ligation chemistry (Reimann et al., 2019).

Recently, a new and rapid ligation method was presented: the additive-free diselenide-selenoester ligation (DSL) (Mitchell et al., 2015). Making use of a peptide selenoester and a peptide diselenide bearing an N-terminal (Sec)_2_ unit, the thiol-free ligation was completed within minutes. Giving an example of the time-saving advantage this method provides, Mitchell et al. were able to completely synthesize an early secretory antigenic protein-6 (ESAT-6) within only 16 h including a deselenization of the Sec unit to Ala (Mitchell et al., 2015). The deselenization reaction is further employable to other amino acids, such as aspartate and glutamate auxiliaries (Conibear et al., 2018) and is even employable to poorly soluble compounds. This is demonstrated by synthesis of the poorly soluble therapeutic lipopeptide tesamorelin and variants of the transmembrane lipoprotein phospholemman FXYD1 using this method and nanomolar concentrations circumventing the integration of solubilizing units (Chisholm et al., 2019). Broadening the ligation toolkit, the α-ketoacid-hydroxylamine (KAHA) ligation route is also employable to poorly soluble and highly hydrophobic proteins such as IFITM3 (see Table 1) or the antibacterial cyclic AS-48 protein (Rohrbacher et al., 2017) making use of 5-oxaproline within acidic conditions. The KAHA ligation is applicable to synthesized fragments from Fmoc-synthesis, integrated solubilizing tags and based on ligation conditions in organic solvents and thus presents an alternative to fragments facing solubility problems.

All presented methods and protocols are based on traditional “batch” chemistry but a novel approach toward continuous-flow peptides synthesis (Mijalis et al., 2017) or ligation and desulfurization (Chisholm et al., 2018). They presented an in-line flow-based ligation and desulfurization protocol and presented synthesis of enfuvirtide (HIV drug) and the diagnostic agent somatorelin. This procedure could be interesting in the future especially considering scale-up of ligation reactions.

Having given an overview of synthetic strategies and follow- up protocols available to date, it is obvious that there is no one-fits-all approach. Membrane proteins are of great interest and thus robust synthesis routes will help to investigate structural behavior leading to a better understanding of the diagnostic points of action and possibilities. Limitations in a straight-forward design are the extreme hydrophobic regions within the protein sequences that lead to aggregation on resin making synthesis challenging. Overcoming this limitation, NCL enables segmentation of the sequence into two or more fragments that can be successively condensed. Especially transmembrane regions are challenging to be synthesized even out of smaller fragments, incorporation of removable solubilizing tags represent a method to facilitate handling, synthesis and purification. However, the general protocols and a variety of choice can be used in order to meet a right choice for the synthesis of any particular “difficult sequences” especially membrane proteins of functional parts of them (Figure 3).

## Author Contributions

LM wrote the first draft of the manuscript. AB prepared the figures and additional references, and wrote parts of the manuscript. HZ read and provided comments for the manuscript, and prepared the tables. AT designed the concept, wrote, revised, corrected, and approved the manuscript. All authors contributed to the manuscript revision, read and approved the submitted version.

## Conflict of Interest

The authors declare that the research was conducted in the absence of any commercial or financial relationships that could be construed as a potential conflict of interest.
